# Molecular Diradical Spin Qubits in a Crystalline Host as a Platform for Quantum Sensing

**DOI:** 10.1021/acscentsci.6c00525

**Published:** 2026-06-29

**Authors:** Sebastian M. Kopp, Jonathan R. Palmer, Brian T. Phelan, Kathryn R. Peinkofer, Shunta Nakamura, Parker A. Watts, Matthew D. Krzyaniak, Michael R. Wasielewski

**Affiliations:** † Department of Chemistry, Institute for Quantum Information Science Research and Engineering, and Center for Molecular Quantum Transduction, Northwestern University, Evanston, Illinois 60208-3113, United States; ‡ Applied Physics Program, 3270 Northwestern University, Evanston, Illinois 60208-3113, United States

## Abstract

Doping a luminescent tris­(2,4,6-trichlorophenyl)­methyl diradical *m* **(TTM)**_**2**_ into a host crystal of its diamagnetic precursor *m* **(HTTM)**_**2**_ creates a molecular color center with enhanced optical-spin interface properties important for quantum sensing. Optical polarization of the |T_0_⟩ sublevel of the diradical triplet ground state is achieved by spin-selective intersystem crossing from the |T_+_⟩ and |T_–_⟩ sublevels of the triplet excited state at ambient and cryogenic temperatures. Coherent spin control of *m* **(TTM)**_**2**_ doped into *m* **(HTTM)**_**2**_ using pulsed optically detected magnetic resonance (ODMR) spectroscopy results in a 10-fold improvement in ODMR contrast over that observed for randomly oriented *m* **(TTM)**_**2**_ using continuous-wave ODMR. The diradical doped crystal powders achieve spin coherence times of 2.8, 3.4, and 7.4 μs at 294, 85, and 5 K, respectively. The diradical photoluminescence is sensitive to weak applied magnetic fields independent of temperature, excitation wavelength, and dopant concentration, providing a promising pathway toward robust quantum sensing of anisotropic magnetic fields under ambient conditions.

## Graphical abstract

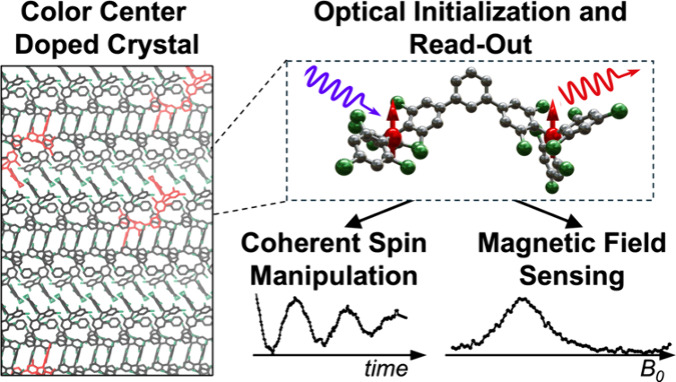

## Introduction

Optically addressable quantum bits (qubits) that can be photoinitialized in a well-defined quantum state, coherently manipulated, and optically read-out hold great promise for quantum information science (QIS) applications, especially quantum sensing.^1–5^ While solid-state defects, such as nitrogen vacancy (NV) centers in diamond, offer spin qubit platforms with a robust optical-spin interface enabling single-qubit initialization, control, and read-out under ambient conditions,^3,6–8^ defect-based qubits lack tunability of their optical and spin properties and suffer from decreasing stability and performance metrics in the metrologically relevant regime near the lattice surface.^9,10^ Therefore, mimicking the NV center optical-spin interface in molecular systems with tunable electronic properties and host matrices is an attractive alternative for bespoke quantum applications.^11–13^ Until recently, molecular NV center analogues have focused on transition-metal complexes that provide qubit tunability through changes in the transition-metal ion, coordination environment, and host matrix with promising prospects for quantum sensing and communication applications.^12,14–18^

More recently, organic molecules with multispin excited or ground states have been explored as tunable optical-spin interfaces that can be spatially positioned with nanoscale precision.^19–27^ Stable organic diradicals that mimic the optical spin polarization and read-out characteristics of NV centers are promising candidates for novel quantum sensing applications by combining the favorable spin relaxation times and biocompatibility of organic molecules with long ground-state spin polarization lifetimes. Luminescent organic radicals such as tris­(2,4,6-trichlorophenyl)­methyl (TTM) are versatile building blocks for molecular color centers because of their tunable absorption and emission properties that can operate in biologically relevant wavelength ranges with high photoluminescence (PL) quantum yields.^28–31^ We recently demonstrated that attaching two luminescent TTM radicals to the *meta*-positions of a benzene bridge (*m* **(TTM)**_**2**_, Figure 1b) constitutes a molecular NV-center analogue that enables optical polarization, coherent microwave control, and optical read-out of its triplet ground state.^32,33^ A similar optical-spin interface has also been demonstrated in a fluorenyl-bridged TTM diradical.^34^

**Figure 1:**
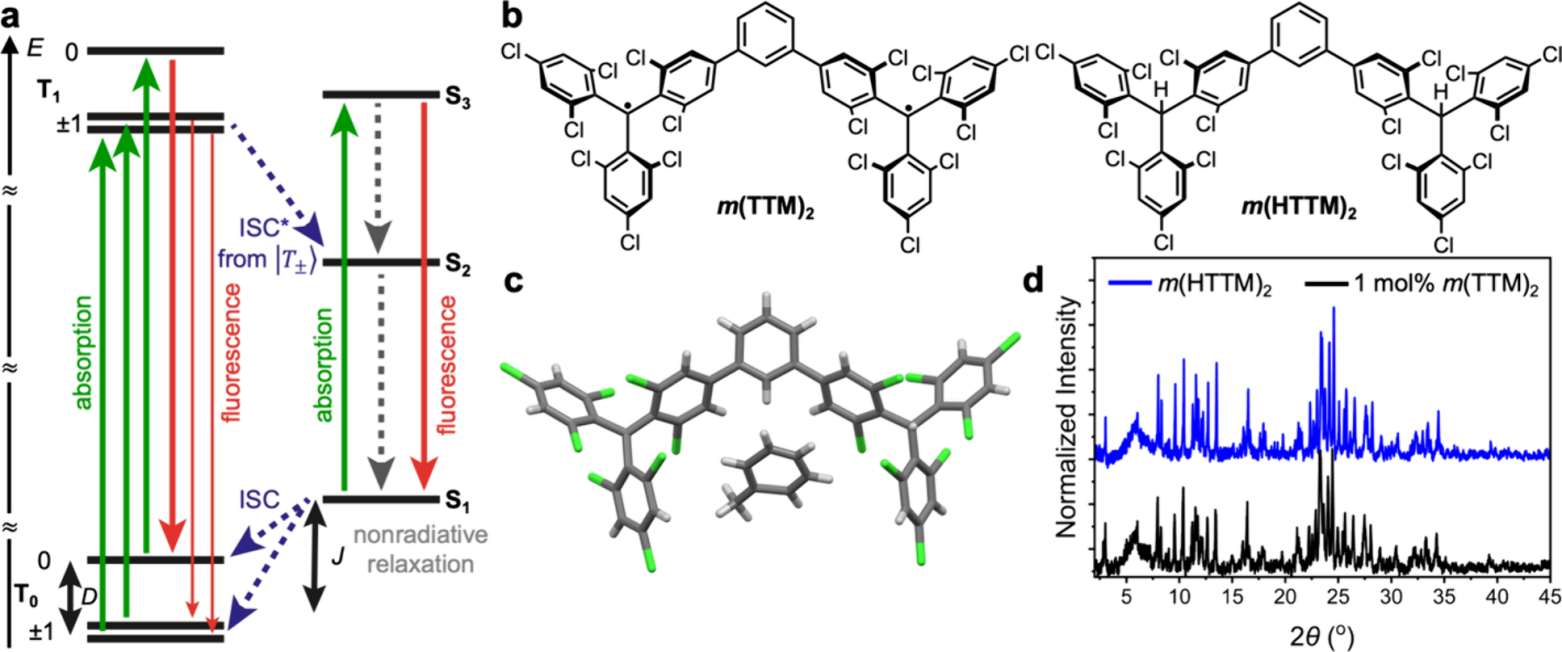
(a) Energy level diagram showing the optical spin polarization and read-out mechanism of luminescent diradical color center qubits. (b) Chemical structures of the organic molecular color center *m* **(TTM)**_**2**_ (left) and the isostructural host material *m* **(HTTM)**_**2**_ (right). (c) Single crystal XRD structure of undoped *m* **(HTTM)**_**2**_. (d) Comparison of the powder XRD spectra of polycrystalline powders of *m* **(HTTM)**_**2**_ (blue) and 1 mol % *m* **(TTM)**_**2**_ doped in *m* **(HTTM)**_**2**_ (black).

The mechanism underpinning the optical-spin interface in *m* **(TTM)**_**2**_ was established computationally^35,36^ and experimentally in dilute frozen solutions in our previous work.^32^ Photoexcitation of the T_0_ → T_1_ transition of *m* **(TTM)**_**2**_ corresponds to the local excitation of one of its TTM subunits. This is followed by a symmetry-breaking intramolecular charge transfer, T_1_ → S_2_, from the photoexcited TTM radical to the ground state TTM radical within *m* **(TTM)**_**2**_, which facilitates spin selective excited-state intersystem crossing (ISC) from the |T_+_⟩ and |T_–_⟩ sublevels of T_1_ to the singlet manifold. This spin selective shelving of triplet populations compensates for the change in orbital angular momentum during the symmetry-breaking intramolecular charge transfer and results in a net-polarization of the triplet |T_0_⟩ sublevel, which can be optically detected by changes in the T_1_ → T_0_ PL intensity (Figure 1a). Relaxation of S_2_ proceeds nonradiatively by charge recombination to the open-shell singlet, S_1_. The optically spin-polarized T_0_ state decays through a combination of nonspin-selective intersystem crossing between S_1_ and T_0_ and spin–lattice relaxation within T_0_.

Here, we demonstrate that doping the *m* **(TTM)**_**2**_ diradical in concentrations ranging from 0.01 to 1 mol % into crystals of its structurally similar precursor *m* **(HTTM)**_**2**_ significantly enhances its optical-spin interface. The excited-state electronic structure and kinetics of these doped crystals were investigated by a combination of steady-state and time-resolved optical spectroscopies confirming high structural ordering and an efficient optical spin-polarization of the ground triplet state analogous to that in NV centers. Electron paramagnetic resonance (EPR) characterization of the triplet ground state shows spin coherence times of up to 2.8 ± 0.2 μs at room temperature and optical polarization of the triplet ground state at both ambient and cryogenic temperatures, which highlights the potential of this optical-spin interface for room temperature applications. Optically detected coherent spin control of the doped crystal powder at 85 K was demonstrated by optically detected magnetic resonance (ODMR) spectroscopy using pulsed light excitation resulting in an ODMR contrast that is more than a 10-fold improvement over what is observed for *m* **(TTM)**_**2**_ randomly oriented in frozen solution using continuous-wave ODMR.^32^ These findings mark an important step toward the use of organic diradicals in nanocrystalline hosts as quantum sensors requiring long spin relaxation times.^8,37–40^ Toward this goal, we demonstrate that the PL of the *m* **(TTM)**_**2**_ diradical doped into *m* **(HTTM)**_**2**_ is sensitive to weak applied magnetic fields independent of temperature, excitation wavelength, and dopant concentration.

## Results and Discussion

### Doped Crystal Characterization

To investigate the performance of *m* **(TTM)**_**2**_ in an ordered solid-state matrix, crystals of the nonradical precursor *m* **(HTTM)**_**2**_ doped with the *m* **(TTM)**_**2**_ diradical were grown by vapor diffusion at room temperature. The diamagnetic precursor *m* **(HTTM)**_**2**_ provides a nearly ideal host lattice for *m* **(TTM)**_**2**_, as both molecules have a similar molecular framework. The only structural difference is the conversion of the central *sp* ^3^-carbon in *m* **(HTTM)**_**2**_ to an *sp* ^2^-carbon in *m* **(TTM)**_**2**_ upon radical formation. This minimal structural change allows for substitutional incorporation of the diradical into the host lattice without introducing significant lattice strain. The excellent agreement between the room-temperature powder XRD patterns of undoped *m* **(HTTM)**_**2**_ and 1 mol % *m* **(TTM)**_**2**_ in *m* **(HTTM)**_**2**_ (Figure 1d) supports this conclusion and indicates that the diradical occupies crystallographically equivalent sites in the host matrix. In addition, the lack of visible-light absorption of *m* **(HTTM)**_**2**_ allows for the selective photoexcitation of *m* **(TTM)**_**2**_ in the doped crystal environment (*vide infra*). Crystals with dopant concentrations of 1, 0.1, 0.05, and 0.01 mol % *m* **(TTM)**_**2**_ in *m* **(HTTM)**_**2**_ were grown to investigate the influence of dopant level on the diradical optical and spin properties. The solution-grown crystals display significant twinning independent of the growth conditions and dopant level. However, small individual flakes with a platelike morphology could be cleaved off for single-crystal XRD (Figure S1). *m* **(HTTM)**_**2**_ crystallizes in a triclinic P-1 space group with two molecules per unit cell (Figure S2). An additional toluene solvent molecule is resolved in the structural refinement, indicating that solvation significantly aids in crystal growth (Figure 1c). *m* **(TTM)**_**2**_ and *m* **(HTTM)**_**2**_ are both *C*_2_-symmetric and consequently chiral, which is consistent with individual molecules in the crystal structure adopting approximate *C*_2_ symmetry. However, as racemic mixtures were used for crystal growth, the sample crystallized into an inversion symmetric point group, which eliminated any potential chirality of the packing.

### Optical Spectroscopy

The steady-state absorption spectra and molar extinction coefficients of *m* **(HTTM)**_**2**_ and *m* **(TTM)**_**2**_ in toluene at 294 K are shown in Figure 2a. *m* **(HTTM)**_**2**_ is colorless and exhibits absorption below 320 nm, whereas *m* **(TTM)**_**2**_ absorbs between 320 and 600 nm, which allows selective photoinitialization of the color center without coexcitation of the host material in the doped crystals. The *m* **(TTM)**_**2**_ doped crystal powders luminesce between 600 and 800 nm at room temperature and 77 K with a sharpening of the spectral features at cryogenic temperatures (Figure 2a). Polarization-resolved steady-state PL spectra of a *m* **(TTM)**_**2**_ doped single crystal were recorded by rotating the linear polarization of the excitation within the plane corresponding to the major face of the plate-like crystal (Figures 2b and S4). The strong linear dichroism confirms the high degree of ordering of *m* **(TTM)**_**2**_ doped into the *m* **(HTTM)**_**2**_ crystal as evident from the narrowly defined angular distribution of transition dipole moments. The room temperature emission kinetics of a *m* **(TTM)**_**2**_ doped crystal powder were measured by time-resolved PL spectroscopy following photoexcitation at 400 nm (Figures 2c and S8). Due to the small ground-state energy gap between the T_0_ and S_1_ electronic states, excitation of *m* **(TTM)**_**2**_ results in local excitation in the singlet and triplet manifolds (Figure 1a). Global kinetic analysis of the time-resolved emission spectra reveals two emissive species with virtually identical decay-associated PL spectra and decay rate constants *k*_em,1_ = (10 ± 2 ns)^−1^ and *k*_em,2_ = (21 ± 2 ns)^−1^. We attribute the fast relaxation process to the deactivation of S_3_, whereas deactivation of T_1_ proceeds on a longer time scale analogous to the cryogenic temperature dilute frozen solution kinetics of *m* **(TTM)**_**2**_.^32^ Importantly, the emission kinetics of *m* **(TTM)**_**2**_ in the *m* **(HTTM)**_**2**_ crystal are independent of the excitation wavelength and dopant concentration within experimental uncertainties (Figure S5–S9). This confirms that PL proceeds from the lowest-energy local excited states and that no intermolecular diradical interactions perturb the excited state lifetimes and spin polarization mechanism in *m* **(TTM)**_**2**_ doped crystals. The low temperature emission kinetics of an *m* **(TTM)**_**2**_ doped crystal powder following photoexcitation at 405 nm at 85 K were measured using time-correlated single photon counting (TCSPC) spectroscopy detected at 600 and 680 nm, respectively (Figure 2d). Global fitting of both TCSPC traces unveils wavelength independent biexponential kinetics with *k*_em,1_ = (10.3 ± 0.2 ns)^−1^ and *k*_em,2_ = (22.8 ± 0.2 ns)^−1^ and wavelength dependent preexponential factors *A*_1_ = 0.42 and *A*_2_ = 0.58 at 600 nm, and *A*_1_ = 0.57 and *A*_2_ = 0.43 at 680 nm. This confirms temperature independent emission dynamics in the singlet and triplet manifolds of *m* **(TTM)**_**2**_ in the solid crystal matrix. Observation of increased singlet emission at longer wavelengths indicates a small bathochromic shift of the singlet emission spectrum analogous to previously reported diradical investigations.^34^

**Figure 2:**
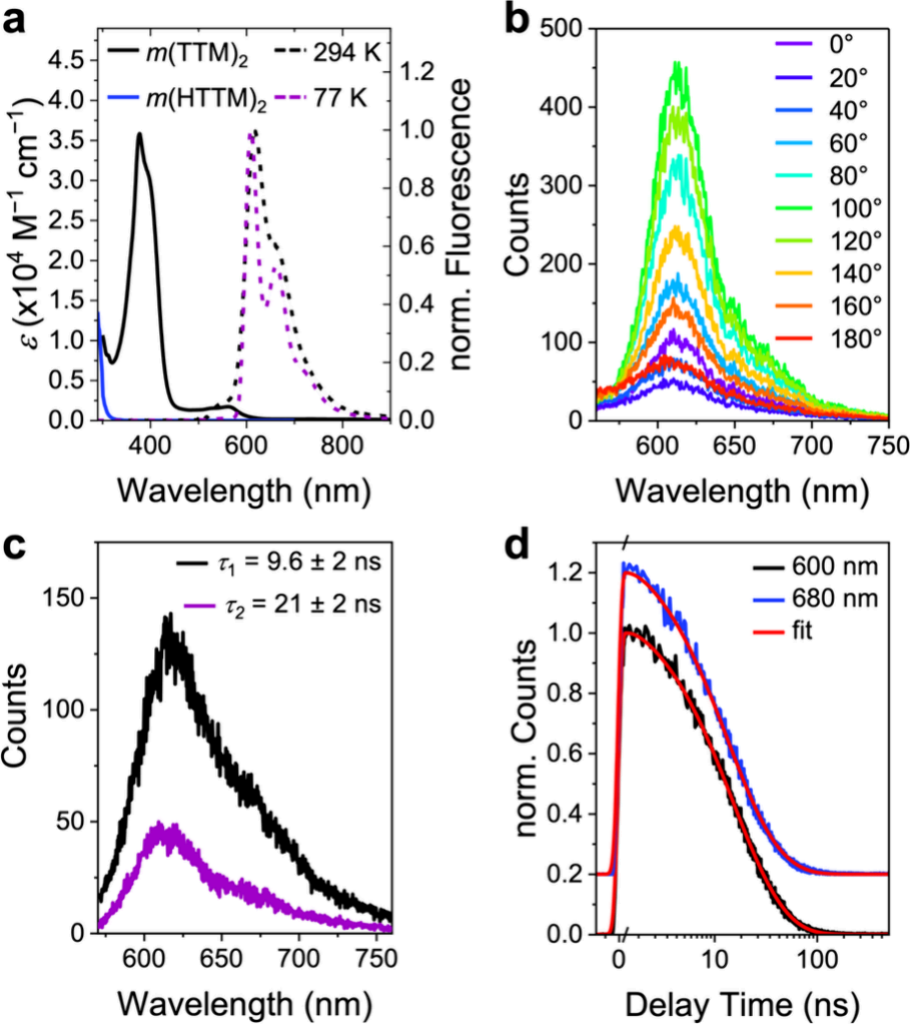
(a) Steady-state absorption spectra of *m* **(TTM)**_**2**_ and *m* **(HTTM)**_**2**_ in toluene at 294 K and normalized steady state fluorescence spectra of *m* **(TTM)**_**2**_ doped crystal powder (0.1 mol %) at 294 and 77 K. (b) Polarization resolved steady state fluorescence spectra of a *m* **(TTM)**_**2**_ doped single crystal (1 mol %). The angles refer to the polarization direction of the linearly polarized excitation light relative to the long axis of the single crystal. (c) Decay-associated spectra obtained via global kinetic analysis of the time-resolved emission spectra of *m* **(TTM)**_**2**_ doped crystal powder (0.1 mol %) following photoexcitation at 400 nm at 294 K. (d) Normalized time-correlated single photon counting (TCSPC) traces of *m* **(TTM)**_**2**_ doped crystal powder (0.1 mol %) at 600 nm (black) and 680 nm (blue) following photoexcitation at 405 nm at 85 K. The TCSPC traces were globally fit with a biexponential decay with decay rate constants and wavelength dependent preexponential factors discussed in the text. The kinetic traces are offset on the vertical axis to aid in visualization.

### EPR Spectroscopy

The magnetic interactions and spin relaxation times of the *m* **(TTM)**_**2**_ triplet ground state in doped crystals were investigated by pulse-EPR spectroscopy at Q-band frequencies. Comparison of the intensity normalized echo-detected EPR spectra of *m* **(TTM)**_**2**_ doped crystal powders as a function of dopant concentration at 85 K shows virtually identical spectral shapes at low dopant concentrations (0.01, 0.05, and 0.1 mol %), whereas increased intermolecular dipolar interactions result in slight spectral broadening in 1 mol % doped crystals (Figure 3a). The echo-detected EPR spectrum of a 0.1 mol % *m* **(TTM)**_**2**_ doped crystal powder was modeled in EasySpin^41^ in the coupled basis of two interacting radicals with Boltzmann sublevel populations (Figure 3b) described by the spin Hamiltonian in eq 1:

$$Ĥ=\mu_{B}g_{1}B_{0}{Ŝ}_{1}+\mu_{B}g_{2}B_{0}{Ŝ}_{2}+2{Ŝ}_{1}D{Ŝ}_{2}$$ 1

where μ_*B*_ is the Bohr magneton, ***B***_**0**_ is the magnetic field vector, ***g***_**1**_ = ***g***_**2**_ are the anisotropic ***g***-tensors of the TTM radical subunits, ***S* ^**_1_ = ***S* ^**_2_ are the electron spin angular momentum operators of the TTM radical subunits, and ***D*** is the dipolar coupling tensor. The factor of 2 in the dipolar coupling term arises from the conversion of the triplet basis to the ***S* ^**_1_, ***S* ^**_2_ coupled diradical basis. We chose this Hamiltonian as a consistent simulation framework for the magnetic resonance spectra in this work to account for the weak spin–spin interactions observed in *m* **(TTM)**_**2**_.^32^ The molecular-frame orientations of the principal *g*- and dipolar-tensor axes were kept consistent with those reported for *m* **(TTM)**_**2**_ in frozen solution.^32^ Simulation of the echo-detected EPR spectrum with the axial and rhombic zero-field splitting parameters |*D*| = 31.7 ± 0.4 MHz and |*E*| = 0 MHz is in excellent agreement with the experimental spectrum (Figure 3b) and confirms weak dipolar interaction in the triplet ground state analogous to previously reported frozen solution investigations of *m* **(TTM)**_**2**_.^32,33^

**Figure 3:**
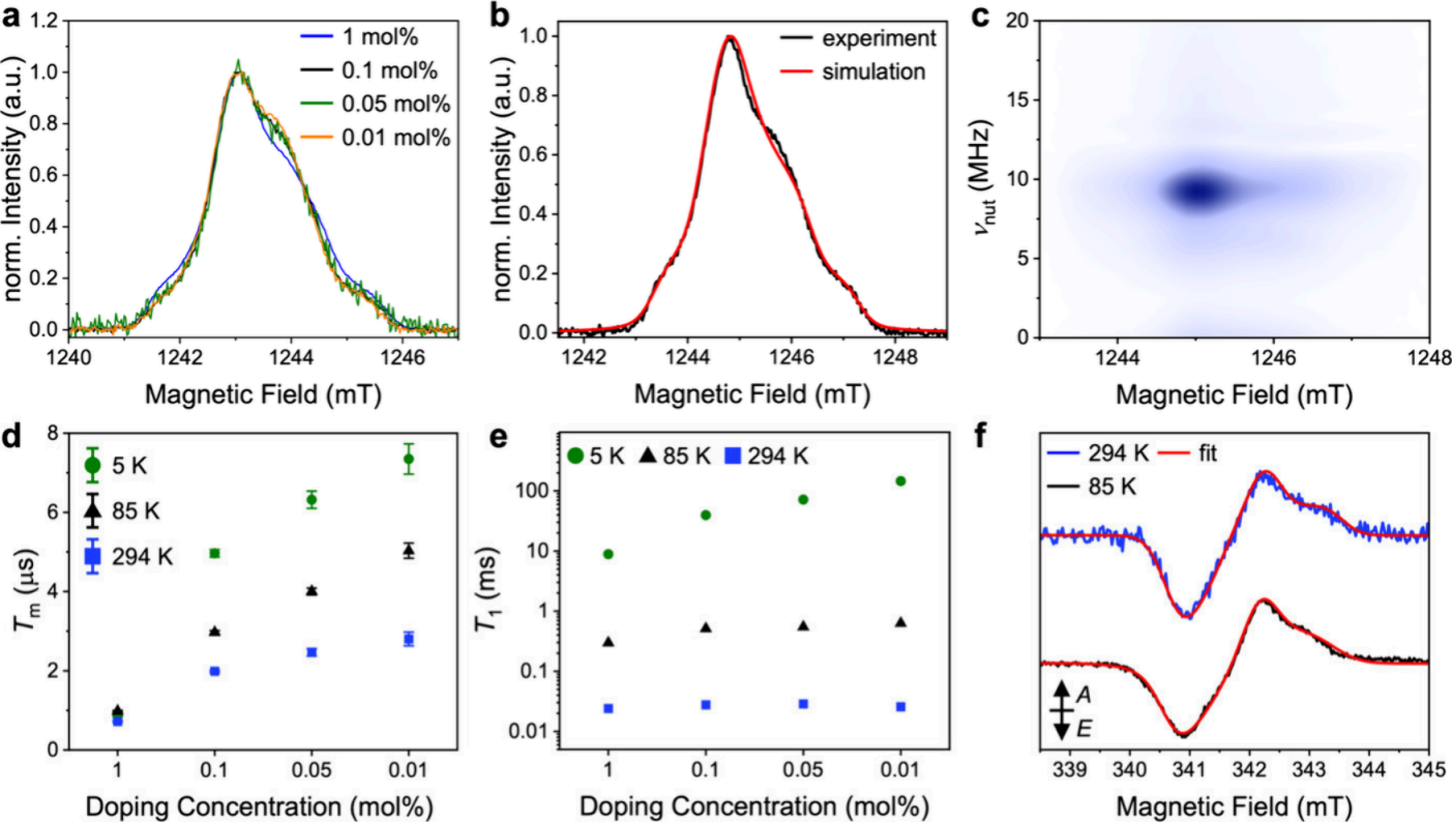
(a) Comparison of the normalized echo-detected EPR spectra of *m* **(TTM)**_**2**_ doped crystal powders with 0.01, 0.05, 0.1, and 1 mol % dopant concentration at 85 K and Q-band frequencies. (b) Experimental (black) and simulated (red) echo-detected EPR spectrum of a *m* **(TTM)**_**2**_ doped crystal powder (0.1 mol %) at 85 K and Q-band frequencies. (c) Fourier transform of the field sweep Rabi nutation experiment of a *m* **(TTM)**_**2**_ doped crystal powder (0.1 mol %) at 85 K and Q-band frequencies. Variable temperature phase memory time, *T*_m_, (d) and spin–lattice relaxation times, *T*_1_, (e) of *m* **(TTM)**_**2**_ doped crystal powders measured as a function of the dopant concentration at Q-band frequencies. (f) Experimental and simulated (red) transient continuous-wave EPR spectra of a *m* **(TTM)**_**2**_ doped crystal powder (0.1 mol %) recorded as an average over 100 ns after the laser excitation at 550 nm at 294 K (blue) and 85 K (black) and X-band frequencies.

The Fourier transform of a three-pulse Rabi nutation experiment as a function of magnetic field of a *m* **(TTM)**_**2**_ doped crystal powder exhibits a single nutation frequency as expected for a paramagnetic ground state with triplet spin multiplicity (Figure 3c). This also confirms the absence of electron Zeeman driven |*S*_0_⟩–|*T*_0_⟩ mixing for *m* **(TTM)**_**2**_, which we attribute to the lack of relative rotations between the individual diradical *g*-tensors within the ordered *m* **(HTTM)**_**2**_ crystal lattice. This confirms that the ordered solid-state environment provided by doped crystals eliminates a substantial decoherence process previously observed in disordered frozen solutions of *m* **(TTM)**_**2**_.^32^ The spin relaxation times of the doped crystal powders were investigated as a function of temperature and dopant concentration at Q-band frequencies. The phase memory times, *T*_m_, increase with decreasing dopant concentration and temperature reaching 2.8 ± 0.2 μs at 294 K and 7.4 ± 0.4 μs at 5 K for the 0.01 mol % *m* **(TTM)**_**2**_ doped crystal powder (Figure 3d). The observation of coherence times of 1 μs or longer at 294 K for all investigated dopant concentrations in a nuclear-spin-rich matrix highlights the potential of these diradical molecular color centers for QIS applications under ambient conditions. Further enhancements of *T*_m_ are expected in single crystal measurements of the *m* **(TTM)**_**2**_ doped crystals due to reduced spectral diffusion in the structurally homogeneous environment. In addition, deuteration of the *m* **(HTTM)**_**2**_ host material is synthetically accessible and offers a straightforward strategy for further extending spin coherence times in future studies. The spin–lattice relaxation time, *T*_1_, of the doped crystal samples determines the upper limit for the polarization lifetime of the photoinitialized triplet ground state. At room and liquid nitrogen temperatures, *T*_1_ is virtually independent of the dopant concentration with *T*_1_ = 27 ± 2 μs and 500 ± 140 μs at 294 and 85 K, respectively. At 5 K, *T*_1_ increases with decreasing dopant concentration from 8.86 ± 0.05 ms for 1 mol % dopant to 146 ± 5 ms for 0.01 mol % dopant (Figure 3e).

Optical polarization of the triplet ground state of *m* **(TTM)**_**2**_ doped into a *m* **(HTTM)**_**2**_ crystal was confirmed by TREPR at 85 and 294 K and X-band frequencies (Figure 3f). The observed *EA* (*E* = emission, *A* = enhanced absorption) polarization pattern confirms a non-Boltzmann population of the triplet ground state due to successful optical initialization of *m* **(TTM)**_**2**_. Observation of photoinitialization over a broad range of temperatures further highlights the potential of these diradical molecular color centers for QIS applications under ambient conditions. Both TREPR spectra were simulated^42^ with identical magnetic interaction parameters (|*D*| = 31.7 MHz, |*E*| = 0 MHz) using the Hamiltonian in eq 1 and an exclusive population of the ground state |*T*_0_⟩ sublevel consistent with the spin polarization mechanism of *m* **(TTM)**_**2**_ (*vide supra*).

### ODMR Spectroscopy

Coherent microwave manipulation and optical read-out of the spin-polarized ground state of a 0.1 mol % *m* **(TTM)**_**2**_ doped crystal powder were achieved by PL-detected magnetic resonance spectroscopy at 85 K (Figure 4). All measurements were performed in the high-field limit at Q-band frequencies to eliminate nuclear spin driven electron spin echo envelope modulation (ESEEM) effects^43^ and to leverage the favorable spin relaxation times of triplet states with small zero-field splitting interactions under strong magnetic fields.^44,45^ The triplet ground state was initialized with a 405 nm laser pulse, followed by the microwave spin manipulation pulse sequence and a laser read-out pulse to detect changes in the PL intensity. Pulsed-light ODMR characterization of the *m* **(TTM)**_**2**_ doped crystal powder yields a 10-fold larger ODMR contrast compared to previous cw-ODMR experiments.^32^ Pulsed optical excitation enables interrogation of a transient highly spin-polarized state immediately following optical initialization and provides an in-depth characterization of the optical-spin interface. In contrast, cw-ODMR probes perturbation of the steady-state spin polarizations under continuous optical excitation, which can reduce the detectable population differences between spin sublevels and reduce the maximum attainable ODMR contrast. The ODMR spectrum is well simulated with the same magnetic interaction parameters employed in the EPR spectral simulations (Figure 4a). The relationship between the experimentally observed ODMR contrast and the extent of ground state polarization was investigated by sweeping the length of the laser initialization pulse, *t*_init_ (Figure 4b) This demonstrates increasing ODMR contrast build-up with increasing pulse length with a maximum ODMR contrast, ΔPL/PL = −0.4% for *t*_init_ = 40 μs followed by a gradually decreasing ODMR contrast with increasing pulse length due to the noticeable onset of spin relaxation processes at longer time scales.

**Figure 4:**
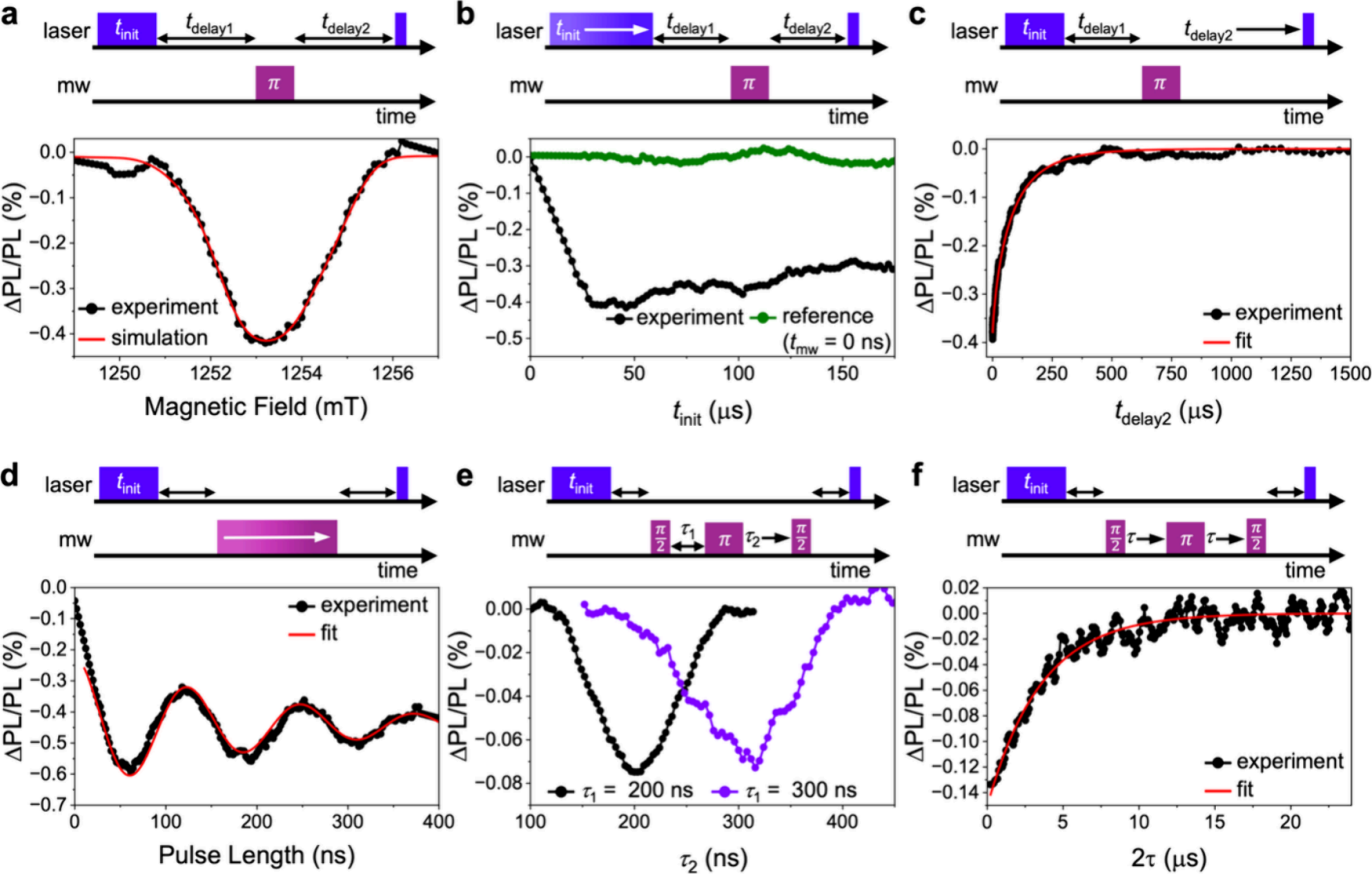
Experimental laser and microwave schemes (top) and fluorescence detected magnetic resonance spectra (bottom) of a *m* **(TTM)**_**2**_ doped crystal powder (0.1 mol %). All measurements were performed at Q-band frequencies and 85 K with photoexcitation at 405 nm. (a) Experimental (black) and simulated (red) optically detected field sweep with *t*_init_ = 75 μs and *t*_delay1_ = *t*_delay2_ = 500 ns. (b) Optically detected spin polarization build-up measurement as a function of optical initialization pulse length with *t*_delay1_ = *t*_delay2_ = 500 ns. (c) Optically detected spin relaxation measurement fit to a monoexponential function with τ = 78 ± 1 μs with *t*_init_ = 70 μs and *t*_delay1_ = 500 ns. (d) Experimental (black) and fitted (red) optically detected Rabi nutation with a Rabi frequency of ∼8 MHz with *t*_init_ = 45 μs and *t*_delay1_ = *t*_delay2_ = 500 ns. (e) Optically detected Hahn echo for τ_1_ = 200 ns (black) and τ_1_ = 300 ns (purple) with *t*_init_ = 45 μs and *t*_delay1_ = *t*_delay2_ = 500 ns. (f) Optically detected phase memory time measurement fit to an exponential decay with *T*_m_ = 3.4 ± 0.3 μs with *t*_init_ = 45 μs and *t*_delay1_ = *t*_delay2_ = 500 ns.

The ground state polarization lifetime was measured by sweeping the delay between the microwave π-pulse and the read-out laser pulse and fitting the data to a monoexponential function with τ = 78 ± 1 μs (Figure 4c). This time is shorter than the *T*_1_ relaxation time measured by EPR under analogous conditions and confirms contributions of nonspin-selective S_1_ → T_0_ intersystem crossing to the decay of triplet ground state polarization. The time scale of S_1_ → T_0_ intersystem crossing in *m* **(TTM)**_**2**_ was previously measured to be 45 ± 3 μs by TA spectroscopy in frozen solution at 85 K,^32^ which is consistent with a shorter effective polarization lifetime. Optically detected coherent microwave control of the spin-polarized *m* **(TTM)**_**2**_ ground state population was demonstrated by driving Rabi oscillations with a maximum ODMR contrast of −0.6% (Figure 4d). The Rabi frequency of approximately 8 MHz confirms that microwave pulses with π/2 and π turning angles are achieved for pulse lengths of 30 and 60 ns, respectively. Coherent control of the electron spin coherences in the crystal is demonstrated by Hahn echo formation using the three-pulse experiment *P*_π/2_ – τ_1_ – *P*_π_ – τ_2_ – *P*_π/2_ – *echo*, where π/2 and π are the microwave pulse turning angles, τ_1_ and τ_2_ are the interpulse delays, and the final π/2 microwave pulse converts the coherence into a population for ODMR observation. Sweeping the second interpulse delay, τ_2_, with a constant first interpulse delay, τ_1_, results in the formation of an approximately Gaussian shaped Hahn echo centered at τ_2_ = τ_1_ (Figure 4e). The coherence lifetime of *m* **(TTM)**_**2**_ was measured in an optically detected Hahn echo decay experiment and fit to a phase memory time, *T*_m_ = 3.4 ± 0.3 μs (Figure 4f). This is consistent with *T*_m_ = 2.97 ± 0.04 μs of the doped crystal powder at 80 K measured by pulse EPR spectroscopy under otherwise analogous conditions (*vide supra*), indicating that the optically polarized and Boltzmann-populated triplet ground states exhibit identical spin coherence times.

### Magnetic Field Sensing

Magnetic field effect (MFE) measurements on *m* **(TTM)**_**2**_ doped crystals reveal an increased PL intensity centered at 0 mT between −4 mT and 4 mT. This effect is temperature, excitation wavelength, and dopant concentration independent and can be observed for doped crystal powders (Figure 5a) and individual single crystals (Figure 5b). Room temperature MFE measurements have also been reported for an analogous TTM-based molecule doped in polystyrene.^34^ We attribute this effect to magnetic field induced triplet sublevel mixing at weak field strengths with a maximum magnetic field induced photoluminescence contrast, ΔPL/PL_0_, of about −2 to −2.5%. This is consistent with the observation of the MFE resonance centered at 0 mT resulting from the small ground-state zero-field splitting in *m* **(TTM)**_**2**_. Perturbation of the PL intensity of *m* **(TTM)**_**2**_ doped crystals by weak magnetic field changes can be utilized for PL-detected magnetic field sensing under ambient conditions without the need for additional microwave manipulations. We hypothesize that single crystal MFE measurements can in principle be used to measure anisotropic magnetic field changes by rotating the principal axis of the zero-field splitting tensor relative to the direction of the magnetic field to be sensed. We are currently pursuing such measurements. Thus, TTM-based diradicals doped into single crystals have potential advantages over such systems randomly oriented in frozen glasses or polymers.

**Figure 5:**
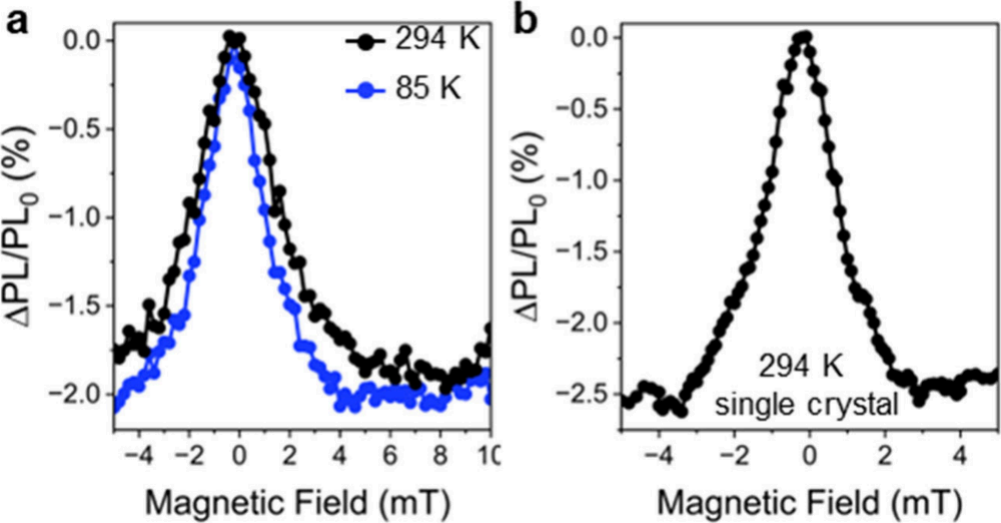
(a) Magnetic field induced fluorescence contrast, ΔPL/PL_0_ = (PL­(*B*) – PL­(*B*_0_))/PL­(*B*_0_), following 405 nm excitation of a *m* **(TTM)**_**2**_ doped crystal powder (0.1 mol %) at room temperature (black) and 85 K (blue), where PL­(*B*) and PL­(*B*_0_) are the fluorescence intensity at an applied magnetic field, *B*, and zero magnetic field, *B*_0_, respectively. (b) Room temperature magnetic field induced fluorescence contrast, ΔPL/PL_0_, following 515 nm photoexcitation of a 1 mol % *m* **(TTM)**_**2**_ doped single crystal.

## Conclusions

Our results demonstrate that TTM-based diradical qubits show enhanced performance as optical-spin interfaces when they are ordered in a crystalline host matrix. This provides a new avenue for the development of quantum sensing materials that leverage the favorable optical and spin properties of luminescent diradicals. Importantly, *m* **(TTM)**_**2**_ doped into *m* **(HTTM)**_**2**_ crystals exhibits optical polarization of the triplet ground state, coherent control of its spin state, and optical read-out under ambient conditions. Optically detected coherent spin control was demonstrated by pulse-excitation ODMR spectroscopy with significantly improved ODMR contrasts and coherence times at 85 K. In addition, *m* **(TTM)**_**2**_ doped crystals were shown to exhibit PL responsive to weak applied magnetic fields at room temperature, making them promising materials for PL-detected quantum sensing of magnetic fields under ambient conditions. We expect that building on these favorable results by performing single crystal experiments with higher laser fluences and optimized triplet spin polarization lifetimes will provide a path toward room temperature pulse-ODMR spectroscopy. Reducing the *m* **(TTM)**_**2**_ dopant concentration further will provide an avenue toward exploring single-molecule spectroscopy of the optical-spin interface.

## Supplementary Material



## Notes

Synthetic procedures, ^1^H and ^13^C NMR, mass spectrometry, additional optical and EPR spectroscopy data, and DFT calculations (PDF)
