# Supplementary material for: Molecular Diradical Spin Qubits in a Crystalline Host as a Platform for Quantum Sensing
Source: ACS Cent Sci. 2026 Jun 29;12(7):1021–8. doi: 10.1021/acscentsci.6c00525 (PMC13397432; doi:10.1021/acscentsci.6c00525)
Supplement: Supplementary file 1 [file oc6c00525_si_001.pdf]

Supporting Information for

Molecular Diradical Spin Qubits in a Crystalline Host as a Platform for Quantum Sensing

Sebastian M. Kopp[1*], Jonathan R. Palmer[1], Brian T. Phelan[1], Kathryn R. Peinkofer[1], Shunta Nakamura[1], Parker A. Watts[1,2], Matthew D. Krzyaniak[1*], and Michael R. Wasielewski[1,2*]

[1]Department of Chemistry, Institute for Quantum Information Science Research and Engineering, and Center for Molecular Quantum Transduction

[2]Applied Physics Program

Northwestern University, Evanston, IL 60208-3113 United States

Email: sebastian.kopp@northwestern.edu); mdkrzyaniak@northwestern.edu; Michael R. Wasielewski (m-wasielewski@northwestern.edu)

Table of Contents

1. Crystal Growth and XRD 2
2. Steady-State Fluorescence Spectroscopy 4
3. Time-Resolved Emission Spectroscopy 4
4. EPR spectroscopy 8
5. Optically detected magnetic resonance spectroscopy 8
6. Fluorescence Detected Magnetic Field Effects 9
7. References 9

1. Crystal Growth and XRD

Synthesis and Purification

Diradical **m(TTM)$_2$** and its precursor **m(HTTM)$_2$** were synthesized, purified, and characterized following our previously reported procedures.[1]

Crystal growth

Crystals of **m(HTTM)$_2$** were grown by vapor diffusion at room temperature. In general, a solution of **m(HTTM)$_2$** in toluene (~5 mg/mL) was prepared and sonicated for 1-2 minutes. Aliquots of this solution (~0.3 mL each) were then pipetted into borosilicate culture tubes (6 × 50 mm). The culture tubes were loaded into a 20 mL scintillation vial filled with ~10 mL of MeOH, then sealed with electrical tape. The solution was left undisturbed until small, white, twinned crystals formed in 1-2 weeks. Crystals of **m(HTTM)$_2$** doped with **m(TTM)$_2$** diradical were grown using the same procedure with slight modifications. Initially, a 45 µM stock solution of the diradical was prepared in toluene ($OD_{560\ nm}$ = 0.015 in a 2 mm cuvette, $\varepsilon_{560\ nm}$ = 1720 $M^{-1}cm^{-1}$). For 1, 0.1, 0.05, and 0.01 mol% diradical dopant, corresponding aliquots of the stock solution (1, 0.1, 0.05, and 0.01 mL, respectively) were added to separate vials each containing 5 mg of **m(HTTM)$_2$**. The resulting solutions were then diluted to total volumes of 1 mL each with additional toluene. Aliquots of these final solutions (~0.3 mL each) were pipetted into borosilicate culture tubes, which were further loaded into 20 mL scintillation vials filled with ~10 mL of MeOH. The vials were sealed with electrical tape and left undisturbed for 1-2 weeks until small, pink, twinned crystals formed. Note that the dopant concentrations given are the mole ratios used for crystal growth and not the exact dopant ratios incorporated into the crystals.

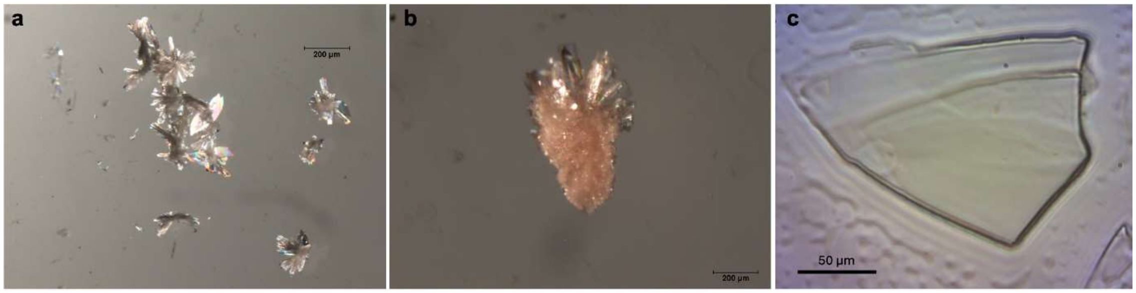

Figure S1. Representative images of undoped ***m*(HTTM)$_2$** (a) and 1 mol% ***m*(TTM)$_2$** doped into ***m*(HTTM)$_2$** (b) crystals exhibiting strong clustering and twinning. (c) Example of a cleaved ***m*(TTM)$_2$** doped crystal flake suitable for single crystal optical spectroscopy.

Single Crystal and Powder XRD

The structure of **m(HTTM)$_2$** crystals was determined using single crystal X-ray diffraction (XRD). Briefly, a single crystal (200 mm × 150 mm × 20 mm) was placed on a MiTeGen loop using paratone oil and mounted in an XtaLAB Synergy diffractometer. The diffractometer was equipped with a microfocus rotating-anode Rigaku Cu X-ray source and a Hybrid Pixel array detector (HyPix). The crystal sample was cooled to 100 K for data collection using an Oxford cryostat. Data reduction, non-merohedral twinning analysis, and empirical absorption correction were performed using CrysAlisPro. The crystal structure was solved in Olex2 software using ShelXT and the intrinsic phasing solution method, then refined using ShelXL and least-squares minimization. The final refined crystal structure was deposited in the

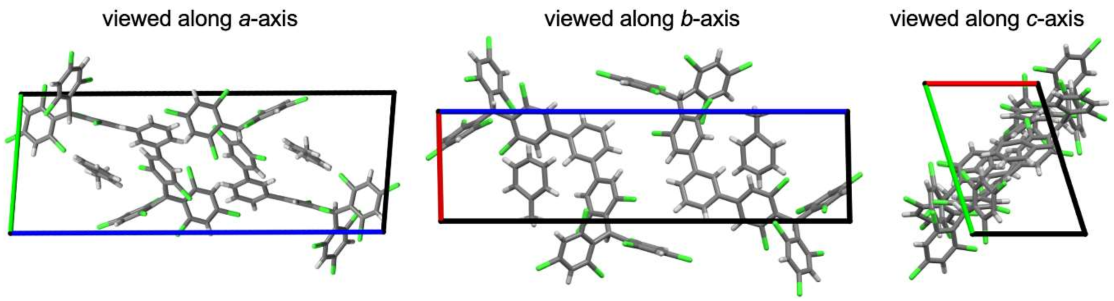

Figure S2. Single crystal packing of undoped ***m*****(HTTM)**$_2$ at 100 K; P-1 space group (a = 8.18 Å, b = 11.49 Å, c = 29.21 Å, α = 84.7°, β = 87.2°, γ = 72.3°); twinned plates (54/46%).

Cambridge Crystallographic Data Center and assigned CCDC number 2504916. Mercury software (version 2022.3.0) was used to visualize all crystal structures.

Powder XRD patterns were collected on samples of undoped and 1 mol%-doped **m(HTTM)**$_2$ that were ground into fine microcrystalline powders. The data were collected at room temperature on a STOE-STADI-P powder diffractometer equipped with an asymmetric curved Germanium monochromator (CuKα1 radiation, λ = 1.54056 Å) and a one-dimensional silicon strip detector (MYTHEN2 1K from DECTRIS). The line focused Cu X-ray tube was operated at 40 kV and 40 mA. The powdered sample was packed in a 3-mm metallic mask and sandwiched between two polyimide layers of tape, which gave a broad, amorphous background signal centered at 2θ ≈ 6° in the diffraction pattern. Intensity data from 2θ = 2-45° were collected over a period of 20 minutes. The instrument was calibrated against a NIST Silicon standard (640d) prior to the measurement. The simulated powder pattern was calculated using Mercury software and the crystal structure of undoped **m(HTTM)**$_2$ as determined from single crystal XRD.

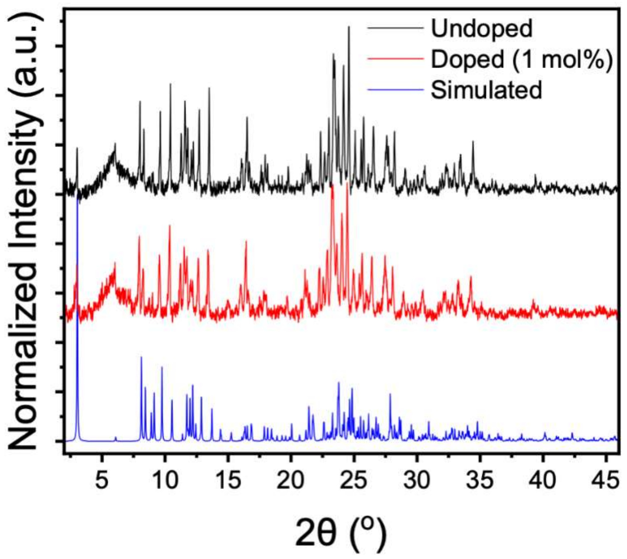

Figure S3. Comparison of the room temperature powder XRD spectra of polycrystalline powders of ***m*****(HTTM)**$_2$ (black) and 1 mol% ***m*****(TTM)**$_2$ doped into ***m*****(HTTM)**$_2$ (red) and the simulated powder XRD structure calculated using the single-crystal XRD structure of ***m*****(HTTM)**$_2$ recorded at 100 K (blue). The broad feature around 7° in the experimental traces arises from the powder XRD sample mask.

2. Steady-State Fluorescence Spectroscopy

Steady-state emission measurements were collected using a Horiba Nanolog Fluorimeter with λ_{ex} = 550 nm at a right-angle geometry. Polarization dependent fluorescence microscopy data were also collected using the Hamamatsu C4334 Streakscope camera system in the analogue integration mode on the Hamamatsu HPD-TA software. A half-wave plate was placed in the pump path to rotate the polarization of the incoming pump. The excitation pulse was tuned to 400 nm. The pump was directed through the back port of a commercial upright optical microscope (Nikon, Eclipse Ti-U) and through a 40x microscope objective (Nikon, CFI Plan Fluor ELWD 40x, 0.6 NA, 3.7-2.7 mm WD) onto a single doped crystal. Emission was collected in the back-scatter geometry to eliminate any inner-filter effects.

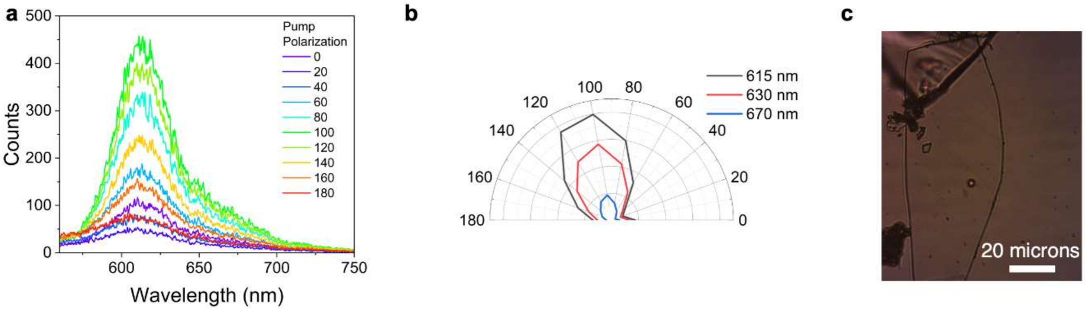

Figure S4. (a) Polarization-resolved, steady-state fluorescence spectra of a ***m*(TTM)$_2$** doped single crystal (1 mol%). The angles refer to the polarization direction of the linearly polarized excitation light within the plane containing the major face of the plate-like crystal. (b) Polar plot of the fluorescence-detected linear dichroism of the ***m*(TTM)$_2$** doped single crystal at selected wavelengths. **c**, Microscope image of the investigated single crystal.

3. Time-Resolved Emission Spectroscopy

Time-resolved fluorescence (TRF) data were measured with a Hamamatsu C4334 Streakscope camera system, and excited using a commercial direct-diode-pumped 100 kHz amplifier (Spirit 1040-HE, Spectra-Physics), producing a 1040-nm fundamental beam (~350 fs, 12 W). The fundamental beam was used to pump a noncollinear optical parametric amplifier (Spirit-NOPA, Spectra-Physics) capable of delivering tunable, high-repetition rate pulses. The excitation pulses were tuned to either 400 nm or 530 nm and passed through a 400 nm or 530 nm band pass filter (Thorlabs, FBH400-10 and FBH530-10) to minimize scatter from the NOPA. The beam was attenuated to < 1nJ/pulse. TRF data were collected via the single-photon-counting mode in the Hamamatsu HPD-TA software using the 100 ns window, with a temporal resolution of approximately 2% of the sweep window (~2 ns). Counts were integrated for ~25 min. The samples were prepared by putting a collection of doped crystals into a 1.8 mm o.d. EPR tube and placing the tube in the focal point of a parabolic mirror. All global analysis fitting was performed using a lab-written MATLAB code. The data was filtered in the spectral domain with a super Gaussian Fourier filter.

Time-correlated single photon counting (TCSPC) measurements were performed by putting a collection of doped crystals into a 1.8 mm o.d. EPR tube, which was placed in an optical access

cryostat (STVP-100 Janis). A 100-mm focal-length lens was used to collect and collimate the emission, which was directed to a fiber-coupled single-photon avalanche photodiode (APD-050-CTE-FC, Micro Photon Devices) paired with a HydraHarp 400 TCSPC system (PicoQuant). A combination of long-pass filters were used to isolated the emission from the scattered pump (515 nm). The emission was coupled into a 2 m fiber optic cable (FG105LCA, Thorlabs) using an achromatic fiber collimator (F950SMA-A, Thorlabs). The TCSPC traces were recorded using the HydraHarp software (PicoQuant) with 65536 channels with 8 ps width. The TCSPC traces were binned in time by a factor of 8 and then globally fit with a sum of exponential functions using the LMFIT nonlinear least-squares minimization package in Python.[2]

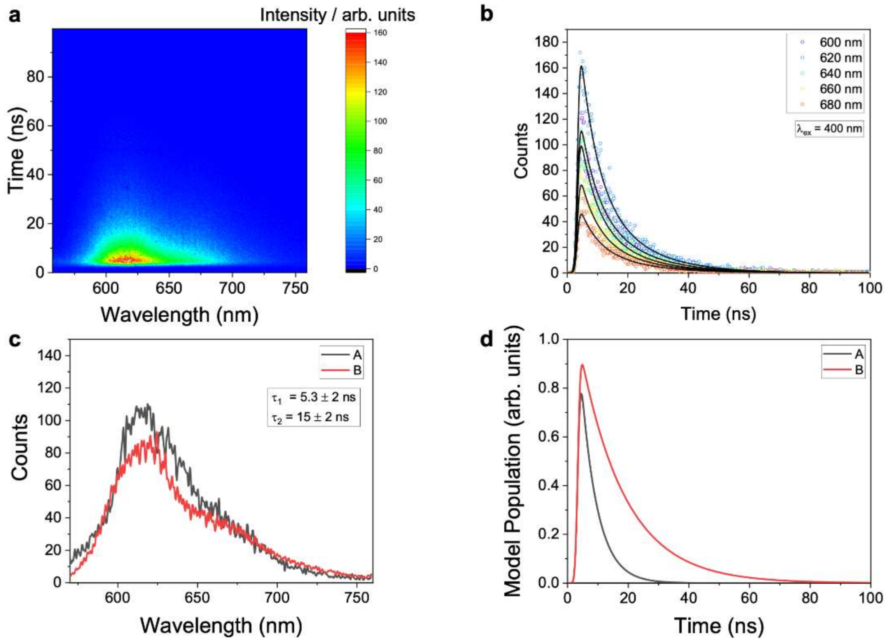

Figure S5. (a) Time-resolved emission spectra of a ***m*(TTM)₂** doped crystal powder (0.01 mol%) at 294 K following excitation at 400 nm. (b) Kinetic traces at select wavelengths overlaid with resulting fits from global kinetic analysis using a biexponential decay model. Corresponding decay-associated spectra (c) and population traces for each state of the kinetic model (d).

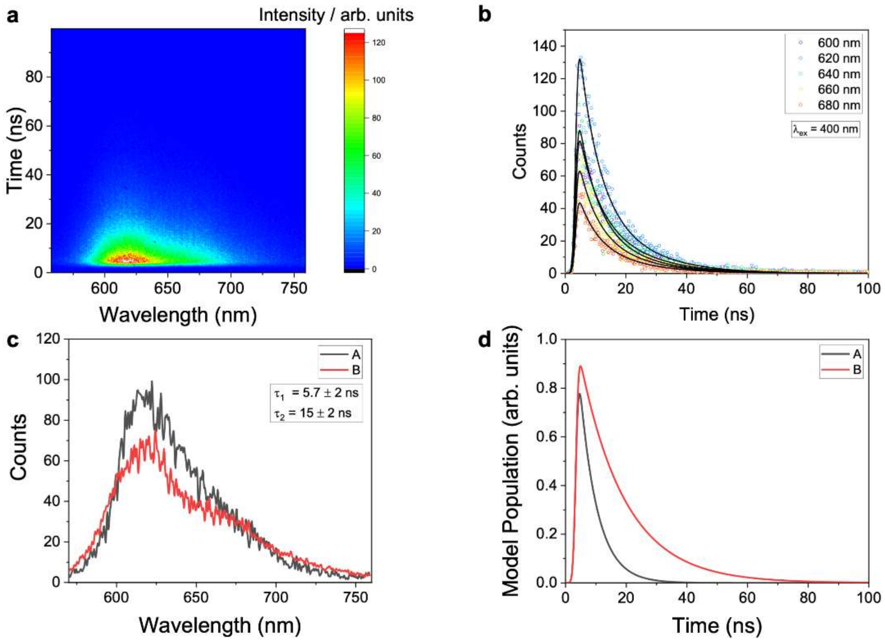

Figure S6. (a) Time-resolved emission spectra of a ***m*(TTM)$_2$** doped crystal powder (0.05 mol%) at 294 K following excitation at 400 nm. (b) Kinetic traces at select wavelengths overlaid with resulting fits from global kinetic analysis using a biexponential decay model. Corresponding decay-associated spectra (c) and population traces for each state of the kinetic model (d).

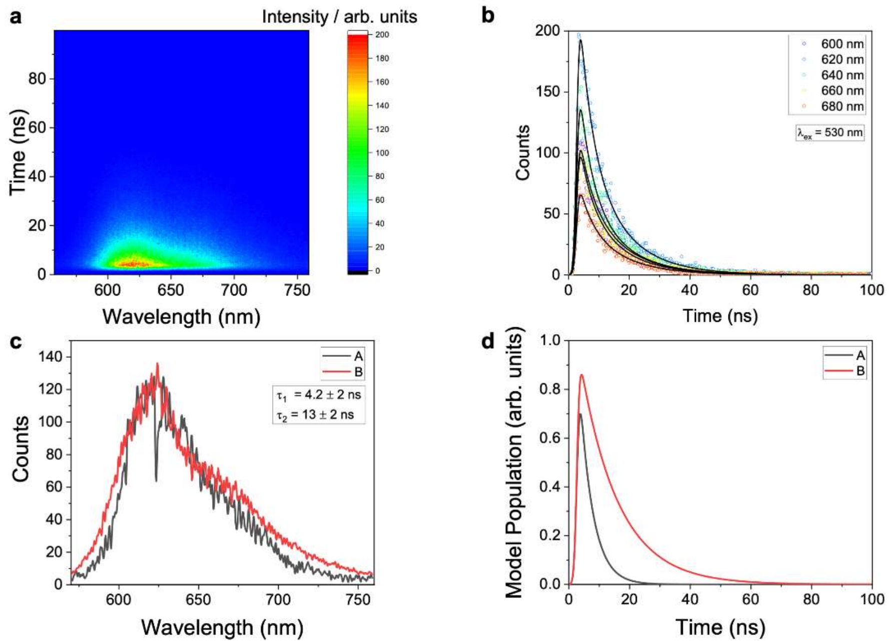

Figure S7. (a) Time-resolved emission spectra of a ***m*(TTM)$_2$** doped crystal powder (0.05 mol%) at 294 K following excitation at 530 nm. (b) Kinetic traces at select wavelengths overlaid with resulting fits from global kinetic analysis using a biexponential decay model. Corresponding decay-associated spectra (c) and population traces for each state of the kinetic model (d).

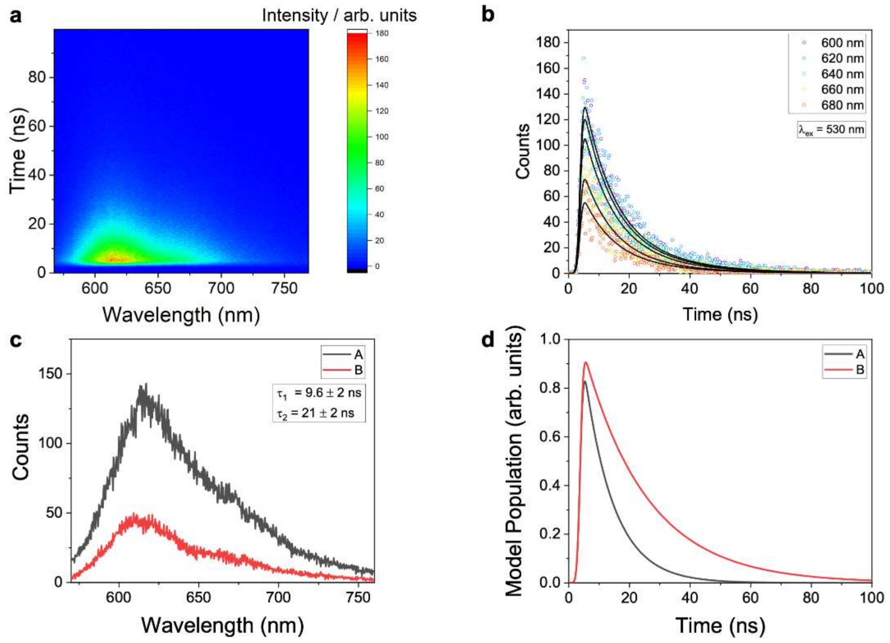

Figure S8. (a) Time-resolved emission spectra of a ***m*(TTM)$_2$** doped crystal powder (0.1 mol%) at 294 K following excitation at 400 nm. (b) Kinetic traces at select wavelengths overlaid with resulting fits from global kinetic analysis using a biexponential decay model. Corresponding decay-associated spectra (c) and population traces for each state of the kinetic model (d).

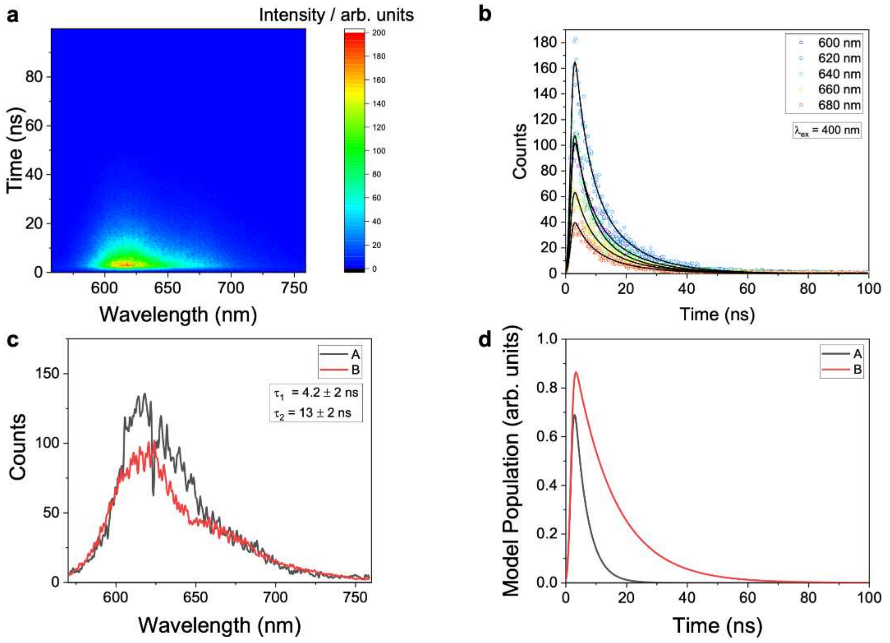

Figure S9. (a) Time-resolved emission spectra of a ***m*(TTM)$_2$** doped crystal powder (1 mol%) at 294 K following excitation at 400 nm. (b) Kinetic traces at select wavelengths overlaid with resulting fits from global kinetic analysis using a biexponential decay model. Corresponding decay-associated spectra (c) and population traces for each state of the kinetic model (d).

4. EPR spectroscopy

The Q-band measurements were performed on a lab-built instrument described previously,[3] but with several changes and modifications, which for completeness will be described. The microwaves were generated with a Keysight M8190A Arbitrary Waveform Generator at an intermediate frequency of between 0.5 and 2.5 GHz and upconverted to 33.2 and 35.2 GHz in a custom Millitech/Smiths interconnect microwave bridge. The bridge outputs maximally ~6 W in high power pulse mode and ~10 mW in continuous wave mode. On the detection end, the signal is first amplified with a Pasternack 22 dB gain low noise amplifier (PEWGA3208) and then down-converted to baseband with a pair of mixers in quadrature, further amplified using a pair of Femto variable gain voltage amplifiers (DHPVA-201) and finally digitized using an Acqiris SA220P DAQ card. The magnetic field was generated using a GMW model 3474-140 magnet and was controlled with a Lakeshore F-41 Gaussmeter. The temperature was controlled using an Oxford Instruments CF935 Cryostat with liquid helium and an ITC503S temperature controller. The experiments were run with resonant frequencies around 34 GHz using a Bridge12 loop-gap resonator (B12TQLP). The instrument was controlled with Specman4EPR a commercially-available control software.[4]

The transient nutation data were collected with a three pulse, $\mathrm{P_{nut} - T - P_{\pi/2} - \tau - P_{\pi} -}$ echo, sequence where: $\mathrm{P_{nut}}$ was a variable length nutation pulse; pulse lengths of $\mathrm{P_{\pi/2} = 35\ ns}$ and $\mathrm{P_{\pi} = 70\ ns}$; and delays $\mathrm{T = 5\mu s}$ and $\mathrm{\tau = 300\ ns}$. Phase memory times were measured with a standard two-pulse, $\mathrm{P_{\pi/2} - \tau - P_{\pi} - echo}$, sequence where: the pulse lengths were $\mathrm{P_{\pi/2} = 30\ ns}$ and $\mathrm{P_{\pi} = 60\ ns}$, and τ was varied. The saturation recovery measurements were collected using a $\mathrm{[P_{sat} - T]_n - T_{rec} - P_{\pi/2} - \tau - P_{\pi} - echo}$ sequence where n=10, $\mathrm{P_{sat} = 45\ ns}$ pulses were applied at $\mathrm{T = 4\ \mu s}$ intervals saturating the EPR spectrum, the recovery after $\mathrm{T_{rec}}$ was measured with a two-pulse echo sequence with pulse lengths of $\mathrm{P_{\pi/2} = 30\ ns}$, $\mathrm{P_{\pi} = 60\ ns}$ and $\mathrm{\tau = 300\ ns}$.

The X-band time-resolved EPR measurements were performed on a Bruker Elexsys E680 X/W EPR spectrometer equipped with a split ring resonator (Bruker ER4118X-MS3). The temperature was kept at 85K using an Oxford Instruments CF935 continuous-flow cryostat cooled with liquid nitrogen and an ITC503S temperature controller. The sample was photoexcited with 7 ns, 0.9 mJ/pulse, 550 nm pulses generated by an optical parametric oscillator (GWU Basi-scan), pumped with the output of a frequency-tripled Nd:YAG laser (Spectra-Physics Quanta-Ray Lab 150). The laser light was coupled into the resonator via a fiber optic (Thorlabs FT1000UMT) and collimator placed outside the cryostat. The transient magnetization was acquired in quadrature under CW irradiation (~2 mW). Sweeping the magnetic field gave 2D spectra versus both time and magnetic field. For each trace, the signal acquired prior to the laser pulse was subtracted from the data. All processing and fitting of the EPR spectra were performed in MATLAB using home written scripts and the simulation package EasySpin v6.0.4.[5-6]

5. Optically detected magnetic resonance spectroscopy

All measurements were performed using the Q-band pulse EPR spectrometer described above. Initialization and read-out laser excitation pulses were delivered by a digitally gated continuous

wave laser (Cobolt 06-MLD, Hübner Photonics) with a nominal cw laser power of 370 mW at 405 nm. The ODMR contrast, ΔPL/PL, was measured using a shot-to-shot differential technique by alternating measurements with and without an applied microwave pulse sequence. The laser excitation was coupled into a fiber optical cable, routed to an optical breadboard mounted in front of the cryostat, collimated using a 25-mm lens, directed to a dichroic mirror, and focused onto the sample using a 50-mm lens. The photoluminescence during the read-out pulse was captured and recollimated with the same lens and transmitted through the dichroic mirror. It was transmitted through a long pass filter, coupled into a fiber optical cable, directed into a gated photomultiplier tube (PMT) (H11526-20-NF, Hamamatsu) and digitized using an Acqiris SA220P DAQ card. The experiment was synchronized using a Keysight arbitrary waveform generator (Keysight M8190A), which triggered a digital delay generator (Stanford Research Systems, DG645) that sent gating TTL pulses to the cw laser and PMT. The AWG and data collection was controlled with Specman4EPR.[4]

6. Photoluminescence Detected Magnetic Field Effects

Sealed quartz tubes with the doped crystal powder were placed in a cryostat (STVP-100 Janis) positioned between the poles of an electromagnet (HV-4W and Magnion HS-735, Walker Scientific). Measurements were performed either under ambient conditions at 294 K or with liquid nitrogen cooling at 85 K. The field strength was measured by a gaussmeter (475 DSP, Lakeshore) with a Hall effect probe. During the experiment, the magnetic field was controlled to $\pm 5 \times 10^{-5}$ T precision. The samples were photoexcited using a digitally gated continuous wave laser (Cobolt 06-MLD, Hübner Photonics) with a nominal cw laser power of 30 mW at 515 nm. The pump was directed to a dichroic mirror and focused on the sample using a 50-mm lens. The photoluminescence was captured with the same lens and recollimated. It was directed through a long pass filter and into a gated photomultiplier tube (PMT) (H11526-20-NF, Hamamatsu) and digitized using a Keysight Agilent U1082A DAQ. The magnetic field control and data collection was controlled with a lab-written LabVIEW program.

Single crystal MFE measurements were conducted inside a closed-cycle optical cryostat (Montana Instruments, Cryostation s50), with crystals held at 294 K while under vacuum ($<10^{-4}$ Torr). The crystals were set on an open-loop piezostage XYZ positioner (Montana Instruments Cryogenic Nanopositioner with an Attocube ANC300 piezo-controller) that was used to locate individual single crystals at the focus of the excitation beam. The crystals were excited using a continuous wave laser (Cobolt 06-MLD, Hübner Photonics) with a cw laser power of 6 mW at 515 nm. The excitation beam was focused into the cryostat via an externally mounted 20x microscope objective (Mitsutoyo, 20x Plan Apochromat objective, 30.5 mm WD, 0.28 NA). The emission was collected in the backscatter geometry with the same 20x objective, separated from the excitation beam with a 550 nm long-pass filter (Thorlabs, FELH550), and directed to a fiber-coupled single-photon avalanche photodiode (APD-050-CTE-FC, Micro Photon Devices) paired with a HydraHarp 400 TCSPC system (PicoQuant) for detection. The magnetic field control and data collection was controlled with a lab-written LabVIEW program.

7. References

1. Kopp, S. M.; Nakamura, S.; Phelan, B. T.; Poh, Y. R.; Tyndall, S. B.; Brown, P. J.; Huang, Y.; Yuen-Zhou, J.; Krzyaniak, M. D.; Wasielewski, M. R., Luminescent organic triplet

diradicals as optically addressable molecular qubits. *J. Am. Chem. Soc.* **2024,** *146*, 27935-27945.
2. Newville, M.; Otten, R.; Nelson, A.; Ingargiola, A.; Stensitzki, T.; Allan, D.; Fox, A.; Carter, F.; Michał; Osborn, R.; Pustakhod, D.; lneuhaus, S.; Weigand, G.; Deil, C.; Mark, A.; Hansen, L. R.; Pasquevich, G.; Foks, L.; Persaud, A., Lmfit/lmfit-py: 1.0.3. *Zenodo* **2021**, https://doi.org/10.5281/zenodo.5570790.
3. Rugg, B. K.; Krzyaniak, M. D.; Phelan, B. T.; Ratner, M. A.; Young, R. M.; Wasielewski, M. R., Photodriven quantum teleportation of an electron spin state in a covalent donor–acceptor–radical system. *Nat. Chem.* **2019,** *11*, 981-986.
4. Epel, B.; Gromov, I.; Stoll, S.; Schweiger, A.; Goldfarb, D., Spectrometer manager: A versatile control software for pulse EPR spectrometers. *Concepts in Magnetic Resonance Part B: Magnetic Resonance Engineering* **2005,** *26B*, 36-45.
5. Stoll, S.; Schweiger, A., Easyspin, a comprehensive software package for spectral simulation and analysis in EPR. *J. Magn. Reson.* **2006,** *178*, 42-55.
6. Tait, C. E.; Krzyaniak, M. D.; Stoll, S., Computational tools for the simulation and analysis of spin-polarized EPR spectra. *J. Magn. Reson.* **2023,** *349*, 107410.
